# An evolutionary algorithm for designing microbial communities via environmental modification

**DOI:** 10.1098/rsif.2021.0348

**Published:** 2021-06-23

**Authors:** Alan R. Pacheco, Daniel Segrè

**Affiliations:** ^1^ Graduate Program in Bioinformatics and Biological Design Center, Boston University, Boston, MA 02215, USA; ^2^ Department of Biology, Boston University, Boston, MA 02215, USA; ^3^ Department of Biomedical Engineering, Boston University, Boston, MA 02215, USA; ^4^ Department of Physics, Boston University, Boston, MA 02215, USA

**Keywords:** microbial communities, synthetic ecology, genetic algorithm, metabolic modelling

## Abstract

Despite a growing understanding of how environmental composition affects microbial communities, it remains difficult to apply this knowledge to the rational design of synthetic multispecies consortia. This is because natural microbial communities can harbour thousands of different organisms and environmental substrates, making up a vast combinatorial space that precludes exhaustive experimental testing and computational prediction. Here, we present a method based on the combination of machine learning and metabolic modelling that selects optimal environmental compositions to produce target community phenotypes. In this framework, dynamic flux balance analysis is used to model the growth of a community in candidate environments. A genetic algorithm is then used to evaluate the behaviour of the community relative to a target phenotype, and subsequently adjust the environment to allow the organisms to approach this target. We apply this iterative process to thousands of *in silico* communities of varying sizes, showing how it can rapidly identify environments that yield desired taxonomic compositions and patterns of metabolic exchange. Moreover, this combination of approaches produces testable predictions for the assembly of experimental microbial communities with specific properties and can facilitate rational environmental design processes for complex microbiomes.

## 1. Introduction

Microbial communities are complex ecosystems that are crucial to the health and function of all biomes, from the oceans to the human gut [1–5]. In addition to yielding a growing understanding of the composition of various microbial ecosystems [6–8], recent advances in DNA sequencing and synthetic biology have enabled new efforts to engineer synthetic multispecies consortia for a variety of applications [9–11]. For example, multispecies systems have been designed to degrade complex substrates or pollutants [12–15], as well as to produce biofuels and molecules for human consumption [15–18]. Advances such as these portend the advent of new applications in synthetic ecology, in which communities of microbes can be readily designed for a vast number of useful outputs. However, this promise is hampered by the difficulty in genetically manipulating individual organisms at community scales, as well as by the lack of a mechanistic understanding of how environmental factors and interspecies interactions shape communities [19–21]. These challenges raise the important question of whether a more accessible parameter, i.e. the chemical composition of the environment, can be modulated to confer specific functions on microbial consortia.

A number of studies have demonstrated the crucial role that changes in environmental composition play in defining microbial community phenotypes, such as in the gut microbiota [22,23] and in aquatic and terrestrial ecosystems [24,25]. As natural ecosystems contain complex combinations of different nutrients, studies have also begun to disentangle the nonintuitive relationship between community properties and resource identity and heterogeneity [24,26–29]. These observations point to the manipulation of environmental composition as a promising method for producing synthetic consortia with defined functions. However, these and other recent studies have demonstrated that community growth and structure can be so sensitive to the environmental composition that even closely related environments can produce very different communities [29,30]. Therefore, in order to reach a phenotype of interest, in practice it often remains necessary to explicitly test a multitude of different specific nutrient combinations—a task that can quickly become experimentally intractable. For example, screening a consortium under all combinations of 20 nutrients—a quantity vastly lower than the number of unique metabolites found in natural settings—would require 1.05 million individual experiments, a scale that remains inaccessible to current conventional microbiological methods. Organism-specific computational models can be deployed to run *in silico* analogues of these experiments [27,31,32], though the number of simulations required would also rapidly become computationally intractable for more complex environmental search spaces.

To begin addressing these challenges, we present here the design of a genetic algorithm (GA) framework to rapidly identify environmental compositions that result in target community phenotypes. This method, conceptually similar to processes used to evolve communities toward specific functions [33–36], searches large spaces of nutrient combinations to produce candidate environmental compositions that optimize specific ecological objectives. Since their inception, GAs have been used widely in the fields of biology and medicine to address a variety of complex optimization problems [37–40]. As they require no explicit knowledge of the underlying dynamics of the biological system being studied, they represent an excellent candidate framework for identifying desired ecological properties in an unbiased way. Nonetheless, applications of GAs to community ecology are rare and have been limited to individual objectives and relatively small combinatorial spaces [41,42]. As such, questions remain as to how they perform in larger search spaces and how algorithm performance can be optimized for a wider variety of community phenotypes.

In order to address these knowledge gaps, we first rely on a large set of *in silico* community experiments consisting of over 6000 unique environment–community pairings. This dataset allows us to identify optimal search parameters and to quantify the performance of our algorithm against known maxima for a variety of objectives. Specifically, we demonstrate the ability of our GA to identify environments that result in desired community compositions, degrees of taxonomic balance and patterns of metabolic secretion and exchange. We then show how this pairing of an evolutionary algorithm with computational models allows us to maximize ecological objectives within a much larger (approx. 600 000 environments) combinatorial space. As our study is limited to *in silico* community data, we also comment on how the methodology presented here can be readily integrated with increasingly available data from ultra-high-throughput experimental platforms, which can produce large sets of community phenotypes in combinatorial environments [43–45]. In sum, this method is able to rapidly identify environmental compositions that optimize a variety of desired microbial community design goals, allowing it to serve as a versatile framework for the exploration of large combinatorial spaces and future applications in experimental synthetic ecology.

## 2. Results

### 2.1. Generation of microbial community phenotypes in combinatorial environments

In order to test our search algorithm, we first simulated the growth of multispecies microbial communities under a large number of environmental compositions. This was done via a dynamic flux balance analysis (dFBA) technique [46] using the COMETS software package [47,48], which enables a mechanistic evaluation of community growth and metabolic exchange using experimentally validated computational models of individual organisms (see Methods). Predictions using dFBA have been shown to recapitulate key microbial phenotypes, while also generating broader statistical mappings of community structure and interactions [27,32,49,50]. Moreover, the use of these models enables the enumeration of a complete set of environment–phenotype mappings that is large yet computationally tractable, allowing us to identify every possible community outcome and evaluate the quality of solutions identified by our algorithm against a known optimum. Our mapping was generated by simulating the growth of 13-species communities in a variety of environmental compositions. The *in silico* organisms that make up our communities were selected as they represent a diverse cross-section of taxa and metabolic capabilities (see Methods), in principle allowing us to maximize the variability of yields, taxonomic compositions and interspecies interactions across different environments. We used combinations of up to 4 of 20 different carbon sources (chosen to limit the large search space) in order to generate a total of 6196 unique environmental compositions. Using COMETS, we inoculated equal amounts of all 13 organisms into these environments and assayed their growth over a simulated 24 h timespan (see Methods).

**Figure 1.**
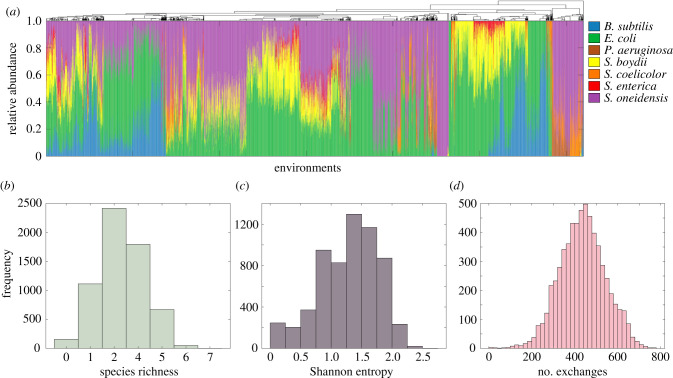
Structural and ecological properties of simulated 13-species communities. (*a*) Relative abundances of organisms after 24 h of growth in all 6196 combinatorial environmental compositions. Only organisms that were present at the end of at least one simulation are shown. Environments are clustered based on species relative abundances (see Methods). (*b–d*)*.* Distributions of species richness (*b*), Shannon entropy (*c*) and the total number of exchanges (*d*) observed across all environments. Here, one exchange is defined as the transfer of a unique metabolite from one organism to another, e.g. the secretion of metabolite *m* by organism *A* and its absorption by organism *B* represents one exchange. As our simulations contained 737 unique extracellular metabolites, the total possible number of exchanges (i.e. if each organism transfers each metabolite to each other organism) totals $\left(\begin{array}{c} 13\\ 2 \end{array}\right)\times 737$, or 57 486.

Our simulated communities displayed high degrees of compositional and functional variability across the environmental conditions we tested (figure 1*a*). At least one organism grew in each environment, and all organisms had stopped growing by the end of the 24 h simulations in all but 40 environments (see Methods). Specifically, six *in silico* organisms (*B. subtilis*, *E. coli*, *P. aeruginosa*, *S. boydii*, *S. coelicolor*, and *S. oneidensis*) reached relative abundances of more than 50% in at least one environment, and all organisms encountered at least one environment in which they could not grow. Organism relative abundances displayed mean variances of 0.02 and species richness values (i.e. the number of organisms present at the end of each simulation) of 3.30 ± 0.99 (mean ± s.d., figure 1*b*). In order to quantify the degree of taxonomic balance in our communities, we calculated the Shannon entropy resulting from each simulation (see Methods). These values were 1.29 ± 0.49 (mean ± s.d., figure 1*c*), which, like our observed relative abundance and species richness values, were comparable to those of similarly sized communities assayed experimentally [29]. We also encountered a wide distribution in the number of metabolic exchanges (defined as the transfer of a unique metabolite from one organism to another) across environments, identifying 435.49 ± 106.49 such transfers per simulation (mean ± s.d., figure 1*d*). We additionally found that that neither our environmental compositions nor the metabolic exchanges observed in our simulations were enough to allow six of the organisms (*K. pneumoniae*, *L. lactis*, *P. gingivalis*, *R. sphaeroides*, *S. cerevisiae* and *Z. mobilis*) to grow, given that they exhibit a variety of metabolic auxotrophies [51–54].

The distributions of these attributes further prompted us to quantify how robust they could be to incremental changes in environmental composition. In doing so, we observed that stronger environmental perturbation generally resulted in more substantial changes to community composition and patterns of metabolic exchange (electronic supplementary material, figure S1). Despite these general trends, however, we observed that even small changes in environmental composition often resulted in significantly different community phenotypes. These observations, along with the diversity of community properties described above, recapitulate elements of the nonintuitive relationship between environment and phenotype observed in nature. As such, they point to our dataset as being a suitable base on which to test our search algorithm.

### 2.2. An evolutionary algorithm rapidly identifies environmental compositions

**Figure 2.**
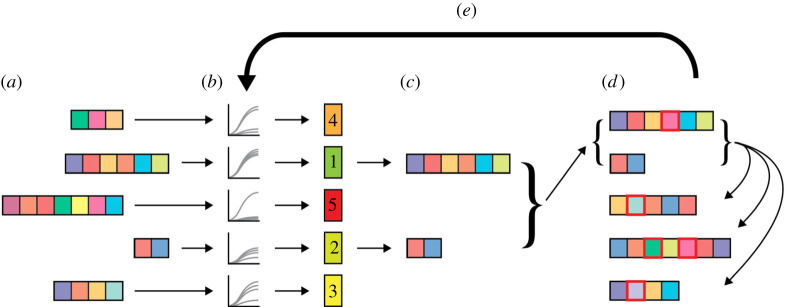
Schematic of GA process for microbial community design. (*a*) A set of *P* environmental compositions, each containing a varying number of limiting nutrients, is randomly generated. (*b*) The community phenotype observed in each environment is determined. As a representative example, this figure shows the GA process with taxonomic balance as the objective to be optimized. The environments are ranked according to their resulting communities' taxonomic balance, and (*c*) the top *σ* environments are selected. Here, the environments that yielded the top σ=2 taxonomically balanced communities are chosen. (*d*) A new population of *P* environments is generated. First, the top *σ* environments are carried over into the new population as ‘parents', and the remaining P−σ ‘offspring' environments are generated via multipoint crossover (i.e. the individual nutrients in the parents are shuffled to produce heterogeneous offspring). Variation is introduced into the new population via mutation, in which each individual element has a defined probability of being changed into a new one (red squares). (*e*) The process of environment ranking, propagation, crossover and mutation is carried out for a total of *G* generations.

Having generated a broad environment–phenotype mapping, we designed a search algorithm to identify environments within this dataset that would result in specific community properties. This method, a GA based on the process of natural selection [55–58], functions as follows: first, a population of *P* environmental compositions is chosen, each containing a random assortment of a maximum of *N* unique nutrients. Community phenotypic data (e.g. species abundances, interspecies interactions and metabolic secretions) on each environment are recorded, and each environment is scored according to the community function being optimized. A subset *σ* containing the top-performing environments is then selected to be propagated to the next generation. The remaining P−σ environments are generated by combining nutrients contained in the top *σ* environments (crossover), and by introducing new nutrients (mutation) at rates defined by a parameter grid search (see Methods; electronic supplementary material, figure S2). The behaviour of the communities on these new *P* environments is recorded, and the optimization process continues until a set of convergence criteria are met (see Methods) or for a maximum of *G* generations (figure 2). The objective of the algorithm is therefore to converge to a final set of environmental compositions that confer the desired properties on the community being tested. For each objective we tested, we also compared the performance of our algorithm to that of a random selection process, whereby new generations were composed of environments randomly selected from the preceding generations (electronic supplementary material, table S3).

**Figure 3.**
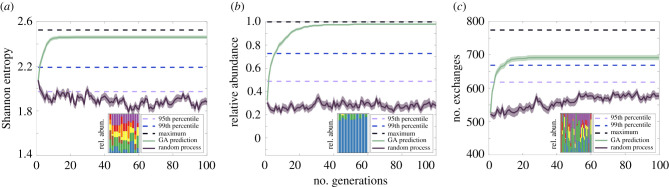
Performance of GA on various ecological objectives. Displayed are the average number of generations (using 50 random seed sets of P=10 environments) required to identify environments that surpassed the 99th percentiles of (*a*) community Shannon entropy, (*b*) the relative abundance of *B. subtilis* and (*c*) the total number of metabolic exchanges between organisms, compared to a random search process. Thick solid lines and shaded regions represent mean and s.e.m., respectively. Insets show the organism relative abundances of the top environmental conditions identified, with colours corresponding to the organisms in figure 1*a*. Performance and convergence plots for each individual seed set of the GA are shown in electronic supplementary material, figure S3. All quantities, including results for optimization of the remaining 12 organisms' relative abundances and performance statistics for the random processes, are found in electronic supplementary material, table S3.

We first applied this framework to identify environments that would maximize the final taxonomic balance of our previously generated communities. Though it is uncommon for organisms to be equally represented in natural settings [59–64], coexistence of multiple organisms is a desirable property for engineered consortia as it can enable tasks useful in biotechnology, such as metabolic division of labour [19,65]. As such, we sought to identify environments within our dataset that resulted in relatively even species abundances. To do this, we applied the GA to search for environments that would maximize the Shannon entropy of our *in silico* communities (see Methods). In order to gain a statistical representation of its performance, we ran our algorithm 50 separate times, each with different random seed compositions of P=10 environments. For each GA process, we recorded the generation at which the algorithm's proposed solutions crossed the 99th percentile of all solutions as a way to quantify its performance. We found that, on average, our algorithm identified solutions that exceeded the 99th percentile of Shannon entropy values after approximately three generations (figure 3*a*; electronic supplementary material, table S3). As each generation tested P=10 environmental compositions, this performance represents explicitly carrying out only 30 unique *in silico* experiments within a space containing 6196 possible nutrient combinations. Though the algorithm generally converged quickly to near-optimal solutions (electronic supplementary material, figure S3 and table S3), we observed variability in the specific environmental compositions it selected. For this particular objective, our method resulted in 13 distinct environmental compositions across the 50 different random seed environments, all of which showed high degrees of consistency and taxonomic balance in the resultant communities (figure 3*a* inset; for specific environmental conditions selected see electronic supplementary material, figure S4).

In addition to optimizing general ecological properties, we tested the capability of our algorithm to identify environments that would maximize more specific features. We first chose to optimize the relative abundances of individual organisms and selected *B. subtilis*, which grew in 2130 out of 6196 environments (figure 1*a*), as a representative example. Again using 50 random initial seed environmental compositions, we found that the GA was able to identify solutions that exceeded the 99th percentile of *B. subtilis* abundances after approximately six generations on average (figure 3*b*). We found that our algorithm selected fewer distinct environmental compositions for this objective across our 50 random seeds, from which 10 distinct environments emerged (figure 3*b* inset). An additional analysis of these environments showed an enrichment for those containing disaccharides (electronic supplementary material, figure S4), pointing to a potential mechanism for maintaining the dominance of *B. subtilis*. Applying our algorithm to the remaining organisms revealed that similarly low numbers of generations were required to reach and converge to optimal solutions (electronic supplementary material, table S3), demonstrating the utility of this framework to identify environments that maximize individual species abundances.

Interspecies metabolic cooperation, often associated with microbial ecosystem stability, is a common target mechanism for community engineering [66,67]. Nonetheless, identifying environments that lead to the emergence of specific interactions remains an elusive task. We thus sought to determine whether our GA framework could also identify desired patterns of metabolic exchange from our computational dataset. We set the total number of interspecies exchanges as our objective function in order to identify the environments that would maximize metabolic cooperation across all organisms. Our GA was able to identify environments that surpassed the 99th percentile of metabolic exchanges after 6.55 generations on average, representing a maximum of 70 *in silico* experiments (figure 3*c*; electronic supplementary material, table S3). Notably, the selected environments resulted in varied taxonomic compositions, ranging from those with high abundances of *E. coli* and *S. oneidensis* to those with more balanced compositions (figure 3*c* inset; for specific environmental conditions selected see electronic supplementary material, figure S4). This result suggests that the degree of metabolic exchange does not necessarily correlate with community taxonomic composition in our dataset, which parallels experimental observations showing conflicting correspondence between taxonomic structure and ecological function [68,69].

Despite its ability to identify environments that exceeded the 99th percentile for these and other optimization targets (electronic supplementary material, table S3), we noticed that some individual runs of the GA were not able to identify the absolute maximum for Shannon entropy and the number of exchanges (figure 3*a,c*; electronic supplementary material, figure S3). This may be due to how these values are distributed (figure 1*c*,*d*), as the maxima are far to the right of the bulk of solutions. Though they may come at a cost to its speed, more stringent criteria can be integrated into the search algorithm if identifying the absolute maximum is desired. Such criteria may be particularly useful for optimization targets that are heavily skewed to the right (electronic supplementary material, figure S5).

Given its ability to optimize the general prevalence of interspecies interactions, we also tested our algorithm on more specific patterns of secretion and exchange. In particular, we sought to determine whether we could identify environments that resulted either in greater metabolic flux toward one particular organism or in the greater overall secretion of a particular metabolite, as such specific phenomena are commonly leveraged for synthetic community design [66,67]. We again used *B. subtilis* as a representative organism to test the former capability, finding that our GA identified environments that surpassed the 99th percentile of metabolic exchanges toward this organism after 9 generations on average. Testing the same capability with our remaining organisms as targets showed similar performance (electronic supplementary material, table S3). We next set the net community-level output of specific metabolites from all organisms as an optimization target, in order to identify environments that would maximize their secretion. To do this, we selected 24 metabolites: 12 that were most highly secreted across all 6196 simulations and 12 that were least secreted. For the former set, we found that while our algorithm identified solutions surpassing the 99th percentile of secretion after 11 generations on average, its performance suffered for metabolites with low secretion flux (electronic supplementary material, table S3).

Despite eventually converging to near-optimal solutions for all of the metabolite secretion patterns we tested, the longer convergence time needed to identify solutions for some metabolites prompted us to quantify its dependence on the number of times a particular metabolite was observed to be secreted across all simulations. We thus analysed the average number of generations needed to surpass the 99th percentile for a given target metabolite with respect to the number of times it was observed in our dataset, finding that these two quantities were inversely proportional to each other (electronic supplementary material, figure S6a). Though this effect reveals a limitation of our method (or indeed of FBA itself), a large number of generations is needed for a rare minority of objectives. For this dataset, we determined that the secretion of 61.4% of organic metabolites could be maximized within 50 generations, with only 21.5% of metabolites requiring over 100 generations (electronic supplementary material, figure S6b).

### 2.3. Searching for community phenotypes in larger combinatorial spaces

Having benchmarked our GA framework on an exhaustive environment–phenotype mapping, we aimed to test its performance in a much larger search space. We thus applied it to determine whether certain environmental compositions could yield communities with highly specific organism relative abundances. This goal draws from efforts to precisely control organism ratios in mixed cultures, which is particularly relevant for synthetic communities applied to the synthesis of biofuels or chemicals [70–72]. Here, we sought to identify environments that would allow one of three organisms—*B. subtilis*, *E. coli* and *S. coelicolor*—to reach a high abundance in a community (90%), while allowing the remaining two to reach low abundances (5% each). We used a list of 154 limiting carbon sources from which we allowed our algorithm to select a maximum of 3, in order to search within a large but well-defined solution space. This search space, consisting of 596 904 unique environmental compositions, remains computationally expensive to test exhaustively using ecological modelling methods like dFBA and nearly impossible to test experimentally. Therefore, this application illustrates the capability of our GA framework to operate in an exploratory fashion within spaces that cannot be fully mapped.

**Figure 4.**
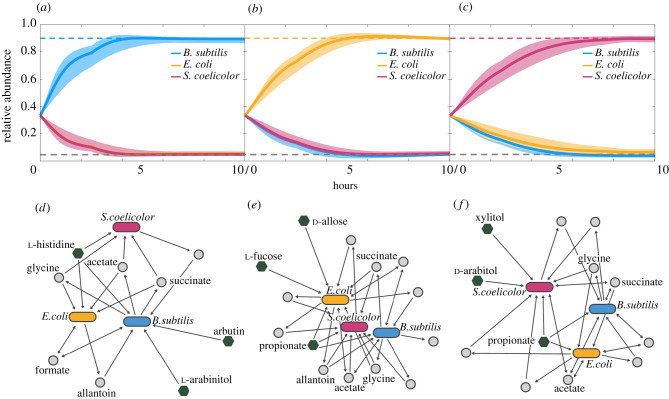
Simulated time-course trajectories of three-species community growth under various GA-determined environments. The GA was used to determine environments that would allow *B. subtilis* (*a*), *E. coli* (*b*) and *S. coelicolor* (*c*) to reach abundances of 90% (upper dashed lines) while the remaining organisms grew to basal levels (5% each, lower dashed lines). Dark lines indicate mean growth curves and shaded regions encompass the maximum and minimum relative abundances for each organism across 10 random environment seed sets. The GA converged to optimal solutions in 10.7 ± 1.4 generations when optimizing for *B. subtilis* dominance, 16.8 ± 7.4 generations for *E. coli*, and 17.6 ± 8.6 generations for *S. coelicolor* (mean ± s.e.m.). (*d–f*) Interaction network structures of representative environments that confer dominance to *B. subtilis* (*d*), *E. coli* (*e*), and *S. coelicolor* (*f*). Elongated ovals represent organisms, green hexagons represent primary nutrients (environmental composition) and grey circles represent exchanged metabolites. Select commonly exchanged metabolites are labelled.

To search this larger combinatorial space, we carried out dFBA simulations of our community in the selected environments as they were produced by the GA, instead of generating a full environment–phenotype mapping *a priori* as above (see Methods). The environments proposed by the GA were scored by calculating the sum squared error between the resulting community compositions and our target abundances [0.90, 0.05, 0.05]. As such, the objective of the GA was to minimize this quantity. We found that, by iteratively searching this large combinatorial space, the GA framework successfully identified environments that allowed each organism to reach a high relative abundance while allowing the remaining two to reach low, but nonzero abundances (figure 4*a*–*c*). Notably, the algorithm converged on multiple such environmental compositions, indicating a type of metabolic flexibility with regard to specific final taxonomic compositions.

We examined the highest scoring environmental compositions in greater detail, identifying common interaction network structures that conferred the desired community phenotypes (figure 4*d*–*f*). For example, in one of the environments that was selected to have *B. subtilis* dominate the community, our dFBA simulation revealed that it was the exclusive consumer of two out of three primary nutrients, while the third nutrient was shared between the three organisms (figure 4*d*). A similar structure was also observed for the environments that optimized dominance of *E. coli* and *S. coelicolor* (figure 4*e*,*f*), as well as in the other compositions selected by the GA (electronic supplementary material, figure S7), suggesting that nutrient specificity was a major driving force of organism dominance in these communities. We also observed dense networks of metabolic byproduct exchange, with molecules such as acetate, formate, glycine and succinate being frequently transferred between organisms, paralleling previous experimental observations of organic acid transfer [73–75]. Given that a crucial element of our objective was for two organisms to reach low abundances, these metabolic exchanges (along with consumption of a third primary nutrient) may be allowing the communities to achieve the desired taxonomic proportions.

## 3. Discussion

The rational design of multispecies communities toward defined phenotypes is an enticing, yet challenging, goal of synthetic ecology. As the phenotypic traits of microbiomes in complex settings remain difficult to predict [29,76,77], fulfilling this potential will require a synthesis of computational and experimental methods that focus on different aspects of these communities [10,78–80]. Here, we used *in silico* microbial communities to show how their ecological properties can be modulated via environmental modification and presented a search algorithm to identify specific nutrient combinations that would result in desired phenotypes. We showed how this algorithm was quickly able to identify high-quality solutions for a variety of ecological objectives: from overall taxonomic balance to specific organism abundances and patterns of metabolic secretion and exchange. Given these capabilities, this method represents a computationally inexpensive way to rapidly screen very large combinatorial spaces to produce desired community properties. Therefore, in addition to optimizing the various objectives tested here, our dFBA–GA framework can be extended to encompass a greater number of important environmental attributes and experimental designs such as varying nutrient concentrations, continuous culture platforms, spatio-temporal nutrient variation and periodic changes in species abundances [47,48,81]. In addition, as genome-scale models can be readily modified to aid in the design of engineered microbial strains [32,82], this framework can serve as a particularly valuable tool for biotechnology applications such as the production of a desired chemical compound.

Despite the flexibility and mechanistic insight afforded by a dFBA approach, engineering synthetic ecosystems *in vitro* will inevitably require experimental validation of modelling predictions. Our approach can be applied to this goal in two ways. First, *in silico* analogues of the desired community may be iteratively screened as we have performed here, and the final environments generated by the GA may then be explicitly tested experimentally. In this way, our method serves as a way to generate an accessible number of testable hypotheses pertaining to specific ecological systems. The pairing of flux balance models and confirmatory experiments in this way has been used extensively to obtain a greater understanding of organism function, as well as to explore previously unknown phenotypes [32,83–86]. However, as high-quality genome-scale reconstructions are limited to relatively few well-characterized model organisms, the applicability of this method is limited to a small set of community taxonomic compositions. Moreover, even these high-quality flux balance models have limitations, which stem from a variety of sources. These include (i) a lack of mechanistic knowledge of reaction-specific uptake rates and kinetic parameters [87,88], (ii) the potential for non-unique FBA solutions and the choice of multiple objective functions [89] and (iii) a limited ability to directly model non-metabolic modes of interaction (e.g. via secondary metabolites) and additional environmental parameters (e.g. pH or temperature) that can impact FBA predictions [87,90].

A second strategy can thus forgo the dFBA component altogether and use the evolutionary algorithm as a way to search through experimentally derived community phenotypic data. As we showed how the GA was able to reach high-quality solutions with relatively few experimental data points, one could envision implementing a similar framework alongside the *in vitro* testing of a community. Here, iterative cycles of testing could be fed into a GA structure, which could inform the next stage of experiments [41,42]. It is in such applications where the structure of a GA becomes particularly relevant, as it is based on testing and producing *populations* of multiple candidate solutions. As such, an experimentally tractable number of candidates can be tested simultaneously, providing a particularly accessible choice of methodology within existing machine learning tools. Moreover, given the transparent nature of the GA's parametrization and search process, it is amenable to a wide variety of parameter choices (e.g. crossover and mutation probabilities) and formulations (e.g. population size and scoring) that can aid its applicability to experimental systems. We therefore propose that, given the increasing accessibility of high-throughput platforms for microbial ecology (e.g. microfluidics, microdroplets, etc.) [43–45], a search algorithm like the one presented here can be deployed alongside such techniques to rapidly reach predefined and complex community objectives.

## 4. Methods

### 4.1. Generation of environment–phenotype mapping with dynamic flux balance analysis

We employed a dFBA method [46] to test the response of a multispecies community in a combinatorial assortment of environments. This process, which was carried out using the COMETS (Computation of Microbial Ecosystems in Time and Space) software package [47,48], allowed us to extract a wide array of phenotypic data from simulated microbial communities. The process by which COMETS carries out these simulations has been outlined in detail in previous publications [27,47,48] and was carried out in the following way for our application. (i) Combinatorial environments were generated by combining an *in silico* minimal medium with limiting quantities of a set of carbon sources. This minimal medium, modelled after the composition of M9, contained nonlimiting concentrations of molecules necessary for growth such as water and ions, as well as sources of nitrogen, phosphorus and sulfur. Limiting amounts of 20 carbon sources were then added on an environment-by-environment basis. These nutrients, an assortment of sugars, organic acids and amino acids (electronic supplementary material, table S2), were added in all combinations of up to 4 at equimolar ratios such that the total concentration of carbon in each environment was 50 mM C in 400 µl. This scheme resulted in 6196 unique environmental compositions. (ii) Genome-scale reconstructions [31,32] of 13 specific microbial organisms were inoculated into our *in silico* media compositions. These organism-specific models span a wide range of taxa and metabolic strategies and were selected to maximize variation in endpoint community composition and interactions across our combinatorial environments (electronic supplementary material, table S1). Based on an approximate total inoculum of OD600 0.05 corresponding to $1.6\times 10^{7}$ cells in 400 µl, and a cell mass of $2.8\times 10^{-13}$ grams dry weight (gDW) [91], all 13 organisms were inoculated into our *in silico* media at equal ratios of $3.45\times 10^{-7}$ gDW for a total inoculum of $4.48\times 10^{-6}$ gDW (OD600 0.05 total). (iii) The growth of these mixed cultures was then simulated in COMETS over the course of 24 h, with a death rate parameter of 0.1 and a timestep of 0.01 h [47]. A more complete list of COMETS modelling parameters is provided in electronic supplementary material, table S5. Once completed, the total final biomass quantities, relative abundances and secreted and absorbed metabolites for each environment were recorded.

To determine whether our communities had stopped growing by the end of the 24 h timespan, we analysed the growth curves of each organism in each environment. If the derivative of the organism's growth curve was greater than zero at least once during the simulation (i.e. the organism grew), and was less than or equal to zero at the end of the simulation, we determined that organism to have stopped growing. For our visualization of the clustered relative abundances of our communities (figure 1*a*), we first computed Spearman correlation coefficients between the species relative abundance vectors under each environment. We then performed hierarchical clustering on these coefficients using the ‘clustergram' function in MATLAB, which calculated distances between clusters using the UPGMA method based on Euclidean distance.

We calculated the Shannon entropy for each community in order to quantify their degrees of taxonomic balance. We define Shannon entropy *H* as

$$H=-\underset{i}{\sum }p_{i}\log_{2}p_{i},$$

where $p_{i}$ is the relative abundance of organism *i* in a community. Organisms in a community with a larger *H* have more equal relative abundances, while those in one with a smaller *H* are less equal, due for example to a single organism outcompeting the rest.

For our second, exploratory application of the GA, a larger pool of 154 carbon sources was used from which a maximum of three nutrients were selected per environment, resulting in 596 904 unique environmental compositions. Here, we did not explicitly simulate the community phenotypes in all combinatorial environments. Instead, only the environmental compositions selected by the GA in each generation were tested and their performance recorded as above. For these simulations, three organism genome-scale reconstructions (*B. subtilis*, *E. coli* and *S. coelicolor* (electronic supplementary material, table S1)) from our list of 13 were used and inoculated into our environments at initial amounts of $1\times 10^{-6}\;\mathrm{gDW}$ each [47]. Additionally, each carbon source was provided at an initial amount of $5\times 10^{-4}\;\mathrm{mmol}$ in order to limit the length of the growth phase. As the goal of this optimization was to allow the three organisms to reach specific relative abundances (as opposed to a longer term test of community stability), we did not integrate a death rate into these simulations (electronic supplementary material, table S5).

### 4.2. Design and parametrization of genetic algorithm

A GA is a search heuristic based on the principle of evolution by natural selection, which optimizes a particular objective function via the modification of a population of individual solutions [56]. Our selection of a GA was based on its applicability to the optimization of nonlinear problems, which reflect the nature of complex environment–phenotype relationships in microbial communities. In our implementation, the individual solutions being modified are unique environmental compositions expressed as vectors denoting the presence of a particular nutrient. The objective function varied according to the phenotype being optimized. In this work, we selected a number of different objective functions to maximize, namely: (i) the overall Shannon entropy of a community as a reflection of taxonomic balance, (ii) the relative abundances of each of the 13 *in silico* organisms, (iii) the total number of metabolic exchanges, (iv) the total metabolic flux directed at each of the 13 *in silico* organisms, (v) the total secretion flux of 24 different metabolic byproducts and (vi) the approximation of target relative abundances. The modifications of different solutions take place over the course of multiple ‘generations', in which each solution is scored according to the phenotype being optimized, and the best solutions are used to seed a new generation of candidate solutions. This process continues with the intent of converging on a set of optimal solutions.

Our implementation of the GA begins with a randomly generated population made up of *P* environmental compositions. In order to demonstrate its extensibility to be used in parallel to an *in vitro* experimental system, we sought to minimize the number of environmental compositions *P* tested in each generation. Therefore, we limited the number of compositions to an experimentally tractable P=10 in each generation. Beginning with this number of environments, the algorithm is initialized and carried out as follows:

1. The *P* environments are initialized with random assortments of up to *N* nutrients (N=4 for our initial benchmarking study, and N=3 for the second exploratory example).
2. The community phenotypes resulting from each environment in the population (pre-generated dFBA data in our benchmarking study, dFBA data generated as needed in our exploratory example, and, in principle, experimental data if being used alongside an *in vitro* system) are recorded and used to assign fitness values to each environment.
3. Each environment is ranked according to the objective function being optimized, and the algorithm selects the top *σ* environments to serve as ‘parents' to the next generation of solutions.
4. Having selected a set of *σ* parent environments, the algorithm uses them to populate a new generation of *P* candidate solutions. This step takes place through processes of crossover (the individual nutrients making up the parent environmental compositions are combined) and mutation (existing nutrients are replaced with new randomly sampled ones). In our implementation, the parent nutrient vectors are linearized, and the remaining P−σ environments are populated with random assortments of the nutrients contained in the parent vector. Mutation then occurs, in which the individual nutrients of all but the top-ranked environment are subject to being randomly replaced by a nutrient yet unused in the current set. The number of environments subject to crossover, as well as the probability of any individual nutrient being subject to mutation, are defined by crossover and mutation probabilities $p_{C}$ and $p_{M}$, respectively (described below).
5. Steps 2–4 are repeated for the new environmental compositions until convergence criteria are met, or for a predetermined number of generations.

We determined optimal values for the crossover and mutation probabilities $p_{C}$ and $p_{M}$ via a parameter grid search. To do this, we selected three representative objective functions: (i) maximization of community Shannon entropy, (ii) maximization of the relative abundance of *B. subtilis* and (iii) maximization of the total number of metabolic exchanges. We then varied the values of $p_{C}$ from 0 to 1 in intervals of 0.1, and the values of $p_{M}$ from 0 to 0.45 in intervals of 0.05. The values of $p_{M}$ were maintained under 0.5 in order to ensure the GA process would not diverge from optimal solutions via excessive mutation. For each pairing of $p_{C}$ and $p_{M}$, we ran our GA 50 times, each with a random seed set of P=10 different environments. We then evaluated the performance of the GA for each objective using a performance score *S*. This score is based on a combination of two metrics: (i) the number of generations required for a set of solutions to surpass the 99th percentile of a given objective $\left(G_{99}\right)$ and (ii) the percentile reached at the final generation of the algorithm $Pr_{\mathrm{end}}$. Since a lower $G_{99}$ denotes better performance, the performance score *S* is defined as follows:

$$S=(1-\tilde{G_{99}})+(\tilde{Pr_{\mathrm{end}}}),$$

where $\tilde{G_{99}}$ and $\tilde{Pr_{\mathrm{end}}}$ are normalized from 0 to 1, such that *S* can range from 0 to 2. We found that the best $\left[\;p_{C},p_{M}\right]$ values were [0.7, 0.25] for our first objective, [1, 0.45] for our second, and [1, 0.4] for our third (electronic supplementary material, figure S2). Interestingly, while our $p_{C}$ values were consistent with commonly used crossover parameter values [92], our calculations revealed low sensitivity of performance scores *S* to changing mutation probabilities $p_{M}$. We thus used an average of the best $\left[\;p_{C},p_{M}\right]$ values ([0.9, 0.35]) for all of our GA objectives.

To determine whether the algorithm has converged to an optimum, we implemented a set of three criteria based on the fitness values of each tested environmental composition. All three of these criteria, based on those previously implemented in evolutionary algorithms [93,94], must be fulfilled in order for the GA to have converged:

1. Populations are internally consistent: the difference between the best fitness and the average fitness within a generation is less than 10% of the average fitness of that generation.
2. Solutions have reached a maximum: the scaled difference in fitness between the best individual in the current generation and the best individual ever discovered is less than 0.01.
3. No further improvement: the fitness scores of the individuals in a generation have not shown a statistically significant increase from those of the preceding generation for at least 10 generations, as determined using a one-tailed *t*-test.

## Data accessibility

All data for our environment–phenotype mapping, as well as scripts for running the GA are available at github.com/segrelab/EvolutionaryAlgorithms. The data are provided in electronic supplementary material.
